# Fast Detection of Missing Thin Propagating Cracks during Deep-Learning-Based Concrete Crack/Non-Crack Classification

**DOI:** 10.3390/s23031419

**Published:** 2023-01-27

**Authors:** Ganesh Kolappan Geetha, Hyun-Jung Yang, Sung-Han Sim

**Affiliations:** 1 School of Civil, Architectural Engineering and Landscape Architecture, Sungkyunkwan University, Suwon 16419, Republic of Korea; 2 Smart Convergence Research Department, Power Technology Research Institute, KEPCO E & C, Gimcheon 39660, Republic of Korea

**Keywords:** deep learning, 1D-CNN, concrete crack and non-crack, thin crack classification, structural health monitoring, fast detection, image processing, UAV, image binarization

## Abstract

Existing deep learning (DL) models can detect wider or thicker segments of cracks that occupy multiple pixels in the width direction, but fail to distinguish the thin tail shallow segment or propagating crack occupying fewer pixels. Therefore, in this study, we proposed a scheme for tracking missing thin/propagating crack segments during DL-based crack identification on concrete surfaces in a computationally efficient manner. The proposed scheme employs image processing as a preprocessor and a postprocessor for a 1D DL model. Image-processing-assisted DL as a precursor to DL eliminates labor-intensive labeling and the plane structural background without any distinguishable features during DL training and testing; the model identifies potential crack candidate regions. Iterative differential sliding-window-based local image processing as a postprocessor to DL tracks missing thin cracks on segments classified as cracks. The capability of the proposed method is demonstrated on low-resolution images with cracks of single-pixel width, captured using unmanned aerial vehicles on concrete structures with different surface textures, different scenes with complicated disturbances, and optical variability. Due to the multi-threshold-based image processing, the overall approach is invariant to the choice of initial sensitivity parameters, hyperparameters, and the sequence of neuron arrangement. Further, this technique is a computationally efficient alternative to semantic segmentation that results in pixelated mapping/classification of thin crack regimes, which requires labor-intensive and skilled labeling.

## 1. Introduction

Rapidly aging civil engineering structures require reliable scheduled maintenance at periodic intervals to increase the safety and longevity of critical load-carrying members. Concrete cracks on the load transfer path grow continuously owing to cyclic loading. Hence, it is critical to identify thin propagating cracks from the perspective of civil engineering structures [1,2,3]. Conventional scheduled maintenance identifies concrete cracks using visual inspection, which requires extensive manual observation and the training of inspectors. To a great extent, the quantification parameters of defects depend on the inspectors’ judgmental skills, which are subjective and prone to error [4]. Hence, there is a need for robust non-destructive inspection (NDI) techniques that result in consistent outcomes for different types of test structures.

NDI inspection methodologies employed by researchers for the inspection of thick structures/concrete comprise computer-vision-based visual inspection [5,6,7,8,9] and physics-based NDI techniques, such as ultrasonics [10,11,12,13], radiography [14], and thermography [15,16,17,18]. Although physics-based NDI techniques are promising for the diagnosis of defects at the coupon level, the complex necessity of tailor-made auxiliary devices for measurement and scalability for in situ field measurements remains questionable. The continuously evolving semiconductor industry affords consumer-grade inexpensive vision-based sensors to NDI inspectors. Computer-vision-based inspection enables rapid large-area measurement scalability with image sensors that are easily configurable with robotic systems [17,19,20,21,22,23] or unmanned aerial vehicles (UAVs) [24,25,26]. Multiple types of autonomous robotic systems, such as ring-type climbing robots, camera-equipped mobile robots, and movable fixtures with multiple degrees of freedom, have recently been employed to explore concrete structures and assist in maintenance [17,19,20,21,22,23,27]. Although robotic-platform-based inspection is a promising way forward for automation and scalability, the following limitations exist: (i) customized systems or auxiliary support systems are required for different structural components and this is often unsuitable for inaccessible surfaces; (ii) robotic platforms often have a short working distance from the target structure to achieve sufficient image resolution for reliable crack detection, i.e., a small field-of-view (FOV), which limits the range of coverage over a specified inspection duration; (iii) although the dimensions of the crack can be accurately identified in image coordinates, a lack of reference or calibration targets within a small FOV results in an erroneous estimation of the true dimensions of the crack in world coordinates; (iv) regarding bridge components, robotic platforms are difficult to operate without additional surface preparation due to the continuous exposure to degrading environmental conditions. UAVs overcome these limitations as they operate at a long standoff distance from a target structure [24]. Although artificial-intelligence-assisted UAVs are promising for covering a large area in a short duration, windy weather conditions in the test field restrict UAVs from flying close to a target structure, thereby reducing the mm/px resolution. In recent years, UAV-based structural assessments have gained considerable attention owing to their enhanced mobility and superior data acquisition capability. Postprocessing, segmentation, and classification of acquired images are often performed using cloud services [26]. Although UAV-enabled edge computing [26] is promising, the performance of cloud computing services limits the efficiency of UAV bridge inspection, and often auxiliary costly onboard edge computing units are installed on UAVs, which is limited to a handful of researchers.

Traditional digital-image-processing-based methods, such as edge detection, morphological operations, digital image correlation, different variants of Otsu’s method, and pattern matching [28,29], are computationally cost-effective. However, they rely on handcrafted selections and predesigned parameters, which hinder automated end-to-end crack detection. Small, shallow, disconnected segments highlighted by conventional digital image processing are often misinterpreted as background noise and require careful handling. In the last decade, researchers have worked on computer-vision-based automated crack inspection for applications in concrete bridge structures [6,30], pavements [31], and buildings [32]. References [33,34] and references therein exhaustively reported the advancement of conventional machine-learning- and deep-learning (DL)-assisted computer vision for automated concrete crack detection. One of the critical challenges in automated crack detection is identifying missing thin/propagating cracks during DL-based concrete crack/non-crack classification.

Close observations of concrete cracks, as reported in the literature [35,36,37], revealed that the thin faint tail of a crack often follows a thick segment of the crack. The reported results indicated a drastic reduction in the detection accuracy for thin cracks with widths of approximately 1–2 px. Concrete cracks propagate over time, and the intrinsic and extrinsic factors near the crack tip determine the propagation rate [38]. The thin tail of propagating cracks often occupies only a few pixels or a single pixel in width, whereas broader or thicker segments of the crack occupy multiple pixels with distinguishable features. The contrast between the thin crack signature and the surrounding background is negligible, and the odds of detecting these features require a microscopic lens with a smaller FOV [24]. For instance, in the case of bridge pier maintenance, as per the standards of ‘Korean bridge inspection and maintenance’, any crack greater than 0.3 mm should be detected. Although state-of-the-art image processing and machine learning techniques have shown promising results in detecting cracks, the reported schemes often fail to detect thin segments of propagating cracks with a single-pixel width. Thus, early diagnosis and maintenance of such thin propagating cracks will increase the corresponding structural lifespan at the component and system levels.

Various image processing schemes and conventional machine-learning-based feature extractions for crack detection are comprehensively discussed in [7,39,40,41,42] and [43,44,45,46], respectively. Irrespective of the choice of state-of-the-art algorithms, the derived results were significantly influenced by field noise in terms of surface textures [47], lighting conditions [48], and shot noise [49]. These image processing and machine-learning-based algorithms are not universal; they are contextual and require a priori information on the potential feature discriminant. Further, although machine learning methods perform well, they require a large number of structured labels. Extracting handcrafted features at crack boundaries to differentiate between crack and non-crack images is challenging [46]. In this regard, supervised DL has proved to be a possible way forward for real-world applications, wherein knowledge of distinct feature extraction is automated. State-of-the-art DL models account for unforeseen scenarios by considering data for different environmental, optical, and structural variabilities. Holistic reviews of AI-based automated surface crack classifications are provided in [34,36,50,51,52,53]. Convolutional neural network (CNN)-based architectures used for crack detection are briefly overviewed in [33]. Every implemented architecture performed well on the available datasets. However, supervised DL with a constant sliding window, bounding boxes, and pixel-level segmentation requires labor-intensive labeling and is computationally costly owing to the deeper layers. Moreover, labeling the ground truth in semantic segmentation is subjective to human expertise. Hence, they are unsuitable for real-time applications implemented using robotic systems or UAVs with limited computational facilities. Although semantic segmentation is widely used for the pixel-level classification of concrete crack features, one of the major limitations is that the detected width of a concrete crack is much wider than the ground truth. This attribute is often associated with a wider width of ground-truth labeling during the training of the DL model to reduce false positives and false negatives.

Recently, researchers have employed a combination of digital image processing schemes and DL for automated crack classification [54,55,56,57]. In [54,56], image processing was employed to assist DL, while [55,57] used DL to assist in image processing. Additionally, Kim et al. [54] used image processing to extract potential crack candidate regions (CCRs) and filter background features, thereby reducing the volume of data required for the DL model. Golding et al. [56] transformed RGB images to grayscale before DL implementation using a pretrained VGG16 architecture, thereby reducing the computation associated with different input channels. Nomura et al. [55] and Yu et al. [57] first extracted bounding boxes of cracks using YOLO-based object detection. Subsequently, the former used a morphological algorithm to pixelate the defect, and the latter used a mask filter to eliminate statistical noise and connected component optimization methods with a global threshold to highlight cracks from the background. Image processing with globally sensitive parameters [55,57] is ineffective for field testing involving UAVs with long standoff distances, where environmental and optical variability are significant, and when the discriminating crack intensity is shallow compared with the background. However, the current state of the literature combining digital image processing schemes and DL still relies on 2D matrix operations during the forward and backward propagation of the DL model.

Despite the significant success of DL, certain segments of thin shallow propagating cracks are often not identified using state-of-the-art DL-based algorithms. However, [58] (i) overcame the limitations of labor-intensive labeling by identifying potential CCRs, wherein image processing was employed as a precursor to the DL model; (ii) identified CCRs to solve the problems associated with big data analysis on a robotic platform; and (iii) achieved computational robustness using a shallow 1D CNN. The potential CCRs in the Fourier basis identified using image preprocessing are fed into the DL model to extract crack information inherently in the frequency domain with a smaller number of neurons and layers. Although state-of-the-art DL shows promising results, it often fails to detect thin propagating cracks with the width of a single pixel in a computationally efficient manner.

The contributions of this study are as follows: To overcome the limitations of state-of-the-art CNNs, pixel-level segmentation, and to utilize the potential of one-dimensional Fourier-based CNNs [58], the authors proposed a scheme considering multi-threshold-based image processing combined with a 1D frequency-domain DL model to detect cracks including thin propagating shallow cracks with a single-pixel width. The proposed scheme tracked missing thin crack segments during DL-based crack identification on a concrete surface in a computationally efficient manner. With image processing, as a preprocessor and postprocessor to the neural network, the overall approach is invariant to the choice of initial sensitivity parameters, hyperparameters, and the sequence of neuron arrangement.

The remainder of this paper is organized as follows: Section 2 discusses the background theories on (i) adaptive-threshold-based integral image binarization for image preprocessing for potential CCR identification and (ii) frequency-domain-based 1D CNN to extract crack discriminant features. In Section 3, an iterative scheme is detailed for tracking thin-propagating cracks with different sliding windows as a postprocessor. In Section 4, we demonstrate the applicability of the developed algorithm for field data from bridge piers. In addition, we discuss the effects of various image-processing parameters and their effects on the predicted results. Finally, we summarize and conclude the study in Section 5 and Section 6, respectively.

## 2. Theory and Method

Here, we concisely discuss the background theory directly used in our proposed implementation scheme: (i) local-threshold-based image processing is used as a precursor to DL to eliminate labor-intensive labeling, and as a postprocessor to track missing shallow propagating crack segments that are often missed by the DL network; (ii) a shallow 1D CNN-based architecture in the frequency domain is adopted to extract discriminant crack features and distinguish cracks in CCRs.

### 2.1. Image Binarization

Although nonlinear neural networks have proven successful in distinguishing crack features, analyzing high-definition (HD) concrete images is computationally expensive when using a deep neural network with multiple cascading layers. The area occupied by the crack or non-crack features in a HD image is generally less than 1%. Local threshold-based intelligent image preprocessing prior to DL enables the identification of discriminant features, such as CCRs, which prevents the background without any structural features from being fed into the DL. Image binarization as a preprocessor eliminates the large area with a plane structural background, reducing the computational burden during the forward and backward propagation of the DL model. Supervised DL techniques require labeled datasets for training, so physically labeling segments of cracks and non-cracks in the presence of a background is cumbersome and prone to human error. Image processing, as a preprocessor, eliminates labor-intensive labeling of the plane structural background and associated human errors. Among various image processing schemes, image binarization effectively filters out background features from discriminant features; image binarization pixelates an RGB image into binary pixel space, and an optimal threshold can be determined that is universally applicable across all sets of training and testing images. Optimal threshold estimation using global approaches based on Otsu’s method [59] and its improvisation [60,61,62] fails to detect crack features when the histogram distribution of pixels in the grayscale (0–255) is unimodal and under non-uniform lighting conditions. However, local thresholding with a sliding window of constant size overcomes these limitations [58,63,64].

**Figure 1:**
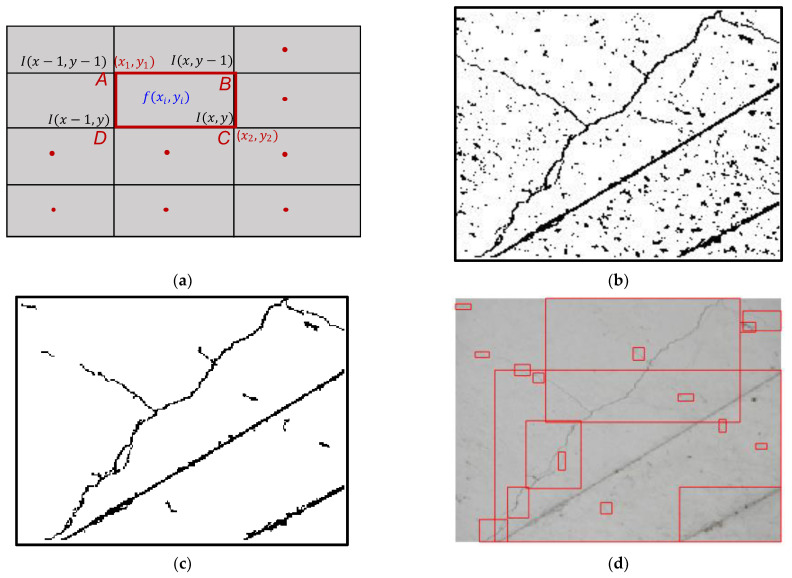
(**a**) Schematic representation for an integral image, and (**b**) results for local-threshold-based image binarization. (**c**) Filtering surface texture and optical noise using aspect ratio of crack, and (**d**) crack candidate regions (CCRs) mapped (marked in red color) to original RGB image.

Below, we briefly discuss the theory of local-adaptive-threshold-based integral image binarization [58,63]. An integral image I(x,y) (called the summed-area table) computed for each pixel (x,y) is given by:

(1) I(x,y)=f(x,y)+I(x−1,y)+I(x,y−1)−I(x−1,y−1)

where f(x,y) denotes pixel intensity at (x,y). A schematic of the integral image computation for each pixel (x,y) is shown in Figure 1a. The cumulative sum over the ABCD region is computed as

(2) $$\begin{array}{cc} f_{s}\left(x,y\right)= & \overset{x_{2}}{\underset{x=x_{1}}{\sum }}\overset{y_{2}}{\underset{y=y_{1}}{\sum }}f\left(x,y\right)\\ & \;\;\;=I\left(x_{2},y_{2}\right)-I\left(x_{2},y_{1}-1\right)-I\left(x_{1}-1,y_{2}\right)+I\left(x_{1}-1,y_{1}-1\right)\; \end{array}$$

For a square window of size s, the local threshold T(x,y) is

(3) $$\begin{array}{c} \;T\left(x,y\right)=\{\begin{array}{c} 1\;\;\;\;\;\;\;\;\;\;\;\;\;\;T\left(x,y\right)>\frac{f_{s}\left(x,y\right)}{s\times s}\times \left(1-\frac{t}{100}\right)\;\\ 0\;\;\;\;\;\;\;\;\;\;\;\;\mathrm{otherwise}\;\;\;\;\;\;\;\;\;\;\;\;\;\;\;\;\;\;\;\;\;\;\;\;\;\;\;\;\;\;\;\;\;\;\; \end{array} \end{array}$$

where T(x,y) is the binarized value and t is the sensitivity factor. Representative results showing binarization involving cracks, non-cracks, and noise are presented in Figure 1b.

Image binarization computes distinct values for each pixel (x,y), which are governed by the local window size and sensitivity factor t (Equation (3)). Equations (1)–(3) can be approximately physically interpreted as the moving average of *s*’ pixels surrounding every pixel (x,y). The adaptive thresholding scheme with a sliding window accounts for spatial illumination variation and thus the approach is invariant to nonuniform lighting conditions. However, a high sensitivity factor introduces many statistical features associated with the surface texture and optical noise [49]; hence, a tradeoff is required to reduce the subsequent analysis. Post-local image binarization (Figure 1b) and connected component analysis [65] extracts a single stretch of pixels without any discontinuity. The pixelated residue was filtered using the aspect ratio of the crack features [54]. A detailed discussion can be found in Ref. [58]. It is impossible to derive a universal value for t that works well for every image. The threshold value is often derived from heuristics that largely satisfy the entire dataset under consideration in concrete structural feature detection. Subsequently, the bounds of the pixelated residue were extracted with bounding boxes that enclose the maximum pixel from left to right and from top to bottom, called CCRs, which include crack and non-crack features. The area of CCRs is not uniform and varies depending on their features. Although the training dataset for DL requires labeling CCRs into cracks and non-cracks, the amount of labeling is significantly reduced compared with up-to-date DL techniques. Figure 1b–d) display the representative results for local image preprocessing and subsequent postprocessing.

### 2.2. Frequency Domain 1D CNN

In the case of a 2D CNN, the kernel slides in 2D data, whereas the kernel slides in 1D for the case of a 1D CNN. This reduces the parameters involved in the DL model and the computational time for training and testing. The advantages of the 1D DL model are as follows: (i) matrix computations associated with gradient-descent-based optimization during forward and backward propagation are replaced with vector array operations; (ii) the 1D DL model learns hidden features with shallow architectures, whereas the 2D DL models require deeper layers to learn hidden features; (iii) 2D DL models often require cloud computing or high-performance GPUs; in contrast, the computational efficacy of 1D CNNs enables their implementation on standard computers, mobile devices, or handheld devices. Although the superior performance of the 1D CNN has been well reported in [44], studies on its application in concrete crack detection are limited to that reported in [58].

**Figure 2:**
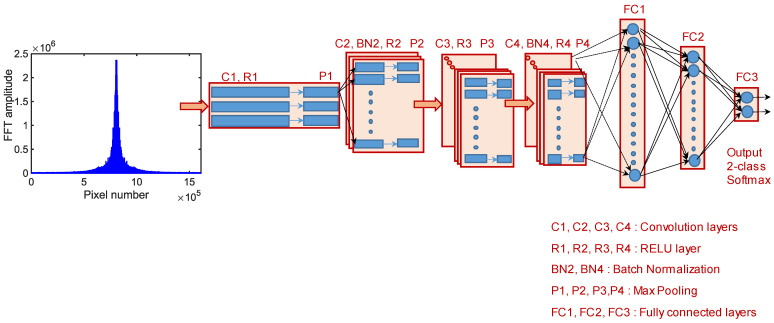
Architecture of 1D discrete Fourier transform (DFT) convolutional neural network (CNN) [58].

Reference [66] reported the potential for crack feature extraction in the frequency domain. Motivated by [66], we input the frequency domain characteristics of CCRs into the DL model [58]. The authors utilized the advantages of distinct crack signatures in the frequency domain for feature extraction in a 1D DL network. This advantage effectively enables a 1D CNN with a shallow architecture and a smaller number of neurons to distinguish cracks from those in the region of interest. The 2D discrete Fourier transform (DFT) (Figure 1a) of an image of size M×N is expressed as:

(4) $$\hat{F}\left(u,v\right)=\frac{1}{MN}\sum_{x=0}^{M-1}\sum_{y=0}^{N-1}f\left(x,y\right)e^{-j2\pi \left(\frac{ux}{M}+\frac{vy}{N}\right)}$$

where f(x,y) is the pixelated image and $e^{-j2\pi \left(\frac{ux}{M}+\frac{vy}{N}\right)}$ is the basis function for each point $\hat{F}\left(u,v\right)$. The complex-valued Fourier transform includes both phase and magnitude information. We performed frequency shifting to transform all the higher magnitudes to the center of the basis system. The transformed 2D pixel space to Fourier basis space information is mutually orthogonal and independent. Subsequently, we vectorized the 2D Fourier space into a 1D space and fed it to the 1D CNN. The 2D CCRs of unequal areas were resized to a uniform size prior to 2D DFT. CCRs greater than or less than 256 px × 256 px were resized or padded with zeros, thereby normalizing the input size for the 2D DFT. The authors implemented a frequency-domain-based 1D CNN for fast classification of surface cracks with a processing rate of approximately 60 images/s [58]. The 1D DFT-CNN was trained on 1492 crack and 1321 non-crack images. The chosen database includes concrete images from different bridge piers that account for the effects of optical variability in terms of lighting conditions, standoff distance between the camera and target structure, focal lengths, FOV, and lens. The configuration of the 1D DFT-CNN-based architecture is depicted in Figure 2. The architecture includes four convolutional layers followed by three fully connected layers. A detailed discussion of the parameters of the architecture can be found in [58]. The optimal number of epochs for training and validation was determined from the asymptotic behavior of the binary entropy loss $L_{BE}\left(y,\hat{y}\right)$, given by:

(5) $$L_{BE}\left(y,\hat{y}\right)=ylog\hat{y}+\left(1-y\right)\log\left(1-\hat{y}\right)$$

where y and $\hat{y}$ are the output and the predicted output, respectively.

## 3. Iterative Postprocessing Scheme for Classification of Missing Thin Propagating Cracks

**Figure 3:**
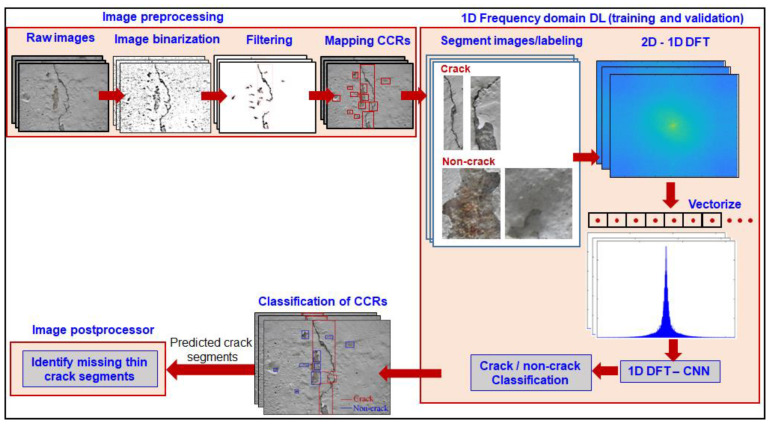
Overall algorithmic scheme for identifying thin propagating cracks, wherein image processing is used as a preprocessor and postprocessor to 1D frequency-domain-based deep learning (DL).

**Figure 4:**
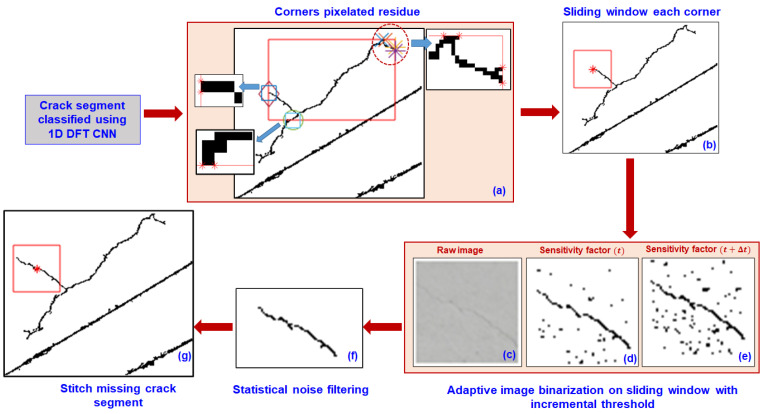
Algorithm for 1D DL-assisted image processing for identification of thin propagating cracks post DL-based crack and non-crack classification. (**a**) Identify endpoints of pixelated residue in the crack segments that the 1D DFT-CNN model classifies (markers indicates the endpoints of pixelated residue; red color rectangle is the bounds of the pixelated residue; circle indicates region of zoom window). (**b**) Define sliding window (red color square) with each endpoint (red star marker) as the centroid of the window. (**c**) Map and extract the corresponding sliding window in the raw image. Adaptive integral binarization on sliding window with (**d**) original sensitivity factor (t) and (**e**) differential sensitivity factor (t+Δt). (**f**) Filtered sliding window using geometric properties of crack. (**g**) Stitch sliding window in pixelated image to connect missing segments of crack.

In the current work, we employed a combination of image-processing-assisted 1D DL and 1D DL-assisted image processing to detect thin propagating shallow cracks. The scheme iteratively uses a differential sliding window, wherein we employ integral image binarization with an increasing threshold to track missing crack segments. The overall framework (Figure 3) for crack identification uses image binarization, first as a preprocessor to the recently developed 1D frequency-domain-based DL by the authors, and finally as a postprocessor to DL to track missing thin shallow cracks with widths less than or equal to a single pixel. This approach combines the advantages of image processing and inherently extracts distinct crack features from a frequency-domain-based CNN. Image processing, as a preprocessor, identifies potential CCRs and significantly reduces the structural background without distinct structural features in subsequent DL-based computations. This enables the replacement of computationally intensive matrix calculations involved in forward and backward propagation in a CNN by a 1D array-based shallow neural network for DL-based hidden feature extraction. Post DL, we performed an iterative scheme with a sliding window of image processing to track thin propagating crack segments, which are often missed by state-of-the-art DL-based schemes (Figure 4). Multi-threshold-based image processing as a precursor and postprocessor provides invariance in the framework to the choice of initial sensitivity parameters during image processing, hyperparameters, and the number of layers in the DL model.

In the overall approach, we first generated CCRs using image processing, wherein adaptive-threshold-based integral image binarization was employed. A large portion of the CCRs arising from shot noise, surface texture, and statistical features associated with image binarization were filtered using the geometric properties of cracks, such as eccentricity and area-based criteria. Next, we mapped the rectangular bounds of filtered CCR segments derived in the binary pixel space to the raw image space (red-colored bounding boxes in the image processing (Figure 3)). Subsequently, the bounding boxes from the raw image were separated from the background and labeled ‘crack’ and ‘non-crack’ for supervised learning. These segmented images of cracks and non-cracks were transformed to a Fourier basis space using 2D DFT and vectorized to 1D to reduce the dimensional complexity. The vectorized DFT was fed as an array input to the 1D DL model. Using the DL model, we extracted discriminant features and classified CCRs as either cracks or non-cracks. The segmented CCRs classified based on the 1D DL model were mapped to the original raw image using the original bounding box coordinates, which were carried forward from image preprocessing to classification CCRs in the original raw image. This approach is sufficient irrespective of the size of the segment. The corresponding classified CCRs were mapped onto the raw images. Although the implementation scheme is similar to the bounding-box-based approach, the outcome is equivalent to semantic segmentation, where we perform pixel-level classification of structural features in a computationally efficient manner by combining image processing with a 1D DFT-CNN. The nonuniform size of CCRs enables scalability, irrespective of the size of the image.

Thin-propagating shallow cracks, which often have widths of approximately a single pixel, are difficult to identify because the distinct features between the crack and surrounding background are negligible. In the scheme for identifying thin shallow propagating cracks, we first identified the endpoints of CCRs that were classified as cracks by the DL model. Although a concrete structure is homogenous, the propagation of cracks at the microscale is governed by the local intrinsic microstructure and extrinsic environmental effects. Hence, we identified all possible endpoints of the pixelated residue (Figure 4a). Next, we constructed a square sliding window spatially in the pixelated binarized domain, with each endpoint as the centroid (Figure 4b). Then, we mapped the corresponding coordinates of the square sliding window from the pixelated binarized domain to the raw image (Figure 4c). We subsequently performed adaptive-threshold-based integral image binarization with an increasing sensitivity factor t+Δt inside the mapped window (Figure 4d,e); a detailed discussion of adaptive-threshold-based integral image binarization is provided in Section 2.1. We filtered out the noise in the sliding window using the geometric properties of the crack (Figure 4f). The thin segment of the shallow crack extracted from the sliding window was connected to the pixelated crack residue obtained from the DL model (Figure 4g). We repeated the procedure using new endpoints of the tracked crack segment. Crack segments are typically wide and deep at the center and gradually become shallow toward their ends. Hence, after each iteration, we increased the threshold used for image processing.

Figure 4 demonstrates the sequence of steps for the 1D DL-assisted image processing. The algorithm sequence was automated without manual intervention. Manual predefined inputs to the algorithm included (i) the size of the sliding window, (ii) incremental sensitivity factor (Δt) for image processing, and (iii) the eccentricity and area threshold to filter the noisy background in the binarized image and texture-related features.

## 4. Results and Discussion of Proposed Scheme on Bridge Images

**Figure 5:**
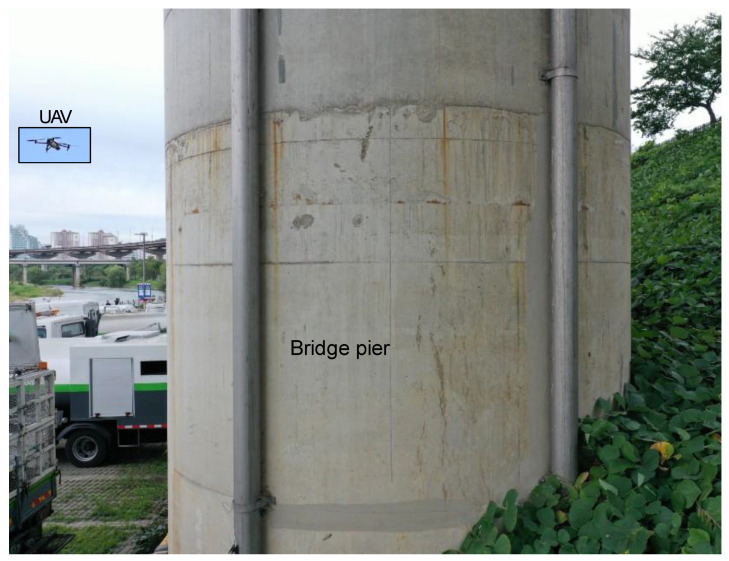
Representative image showing unmanned aerial vehicle (UAV)-based prototype used for bridge pier inspection.

**Figure A1:**
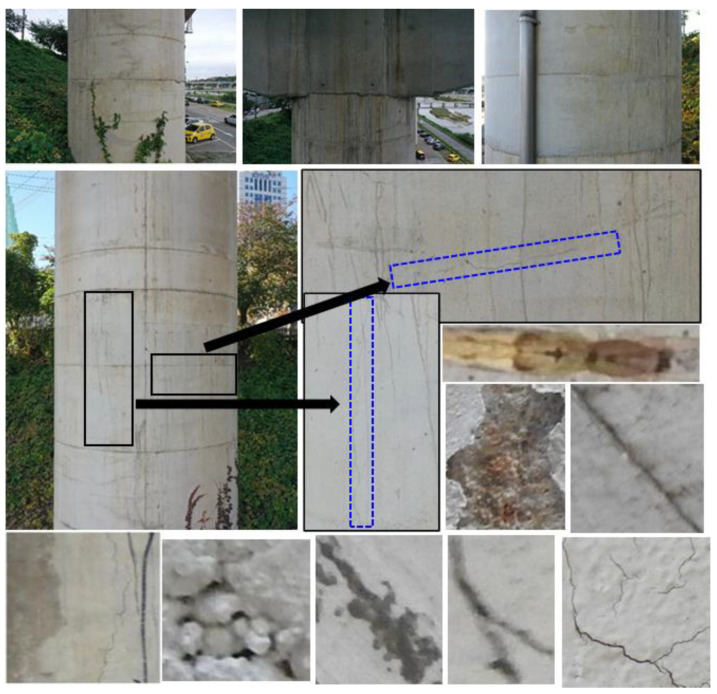
Representative images obtained from UAV for inspection of the bridge pier.

In this study, we employed a UAV to inspect bridge piers. The UAV prototype is a highly stable, cost-effective, and off-the-shelf quadcopter that can be dynamically controlled using a smartphone-based interface. The UAV has a built-in stabilizer with an inertial unit system and a gyroscope. The prototype is equipped with a sensing and communication module, a camera that records HD images, an inertial measurement system for acceleration, a distance measurement module, and a Wi-Fi module that provides a connection between the UAV-based system and the remote system of the operator. Remote access to a UAV-based system allows dynamic control, updated position/distance information, and instantaneous data/image transfer. The cameras were mounted on a gimbal platform that enabled the operator to instantaneously adjust and monitor the video and images acquired by the camera. The camera was equipped with a 1/2” CMOS image sensor to obtain an RGB pixel image of 3648 px × 5472 px, with the maximum operating FOV of 15°. Because of safety concerns regarding the wind-induced impact of the UAV on the target bridge pier, we operated the UAV at a long standoff distance, typically in the range of 3–4 m from the target structure; hence, the resolution per pixel was approximately 0.8–1 mm/px. Often, the width of a thin propagating crack is less than a pixel, and the crack characteristics are indistinguishable compared with the background, which adds to the complexity of the problem, where bridge maintenance necessitates the detection of crack widths of sizes greater than or equal to 0.3 mm. However, a distinct crack signature is recorded at a pixel provided that the crack signature is greater than half the width of the pixel resolution. A representative image of the UAV-based prototype employed for bridge pier inspection is presented in Figure 5. Sample images obtained from the UAV for the inspection of the bridge pier are shown in Figure A1. The database includes crack-like features and complicated disturbances, such as structural edges and markings, concrete peeling, surface stains, and spider stains. The chosen database of concrete images of bridge piers accounts for the effects of optical variability in terms of lighting conditions, standoff distance between the camera and target structure, focal lengths, FOV, and lens. In addition, we considered concrete surface texture variability by populating a database with images from different bridge piers. The proposed model was trained and tested using this broad database, thereby accounting for environmental uncertainty and noise-induced effects.

**Figure 6:**
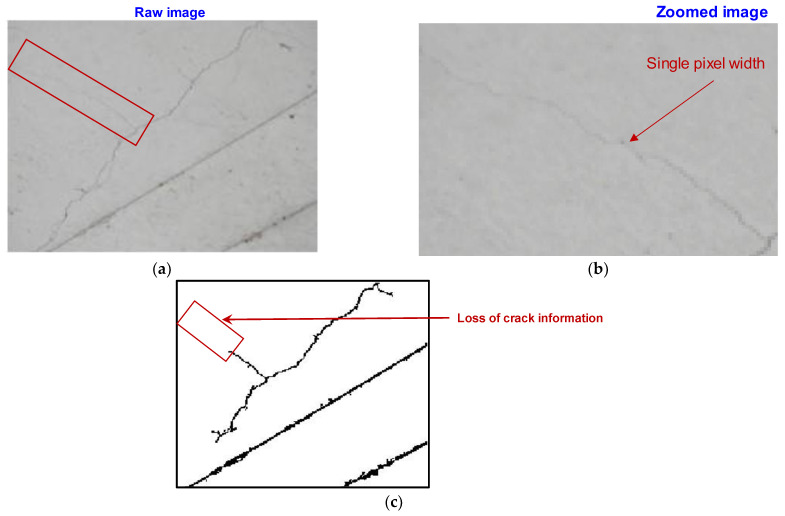
(**a**) Representative raw image and (**b**) zoomed region showing single-pixel width crack. (**c**) Loss of crack information during noise filtering post adaptive-threshold-based integral image binarization.

**Figure 7:**
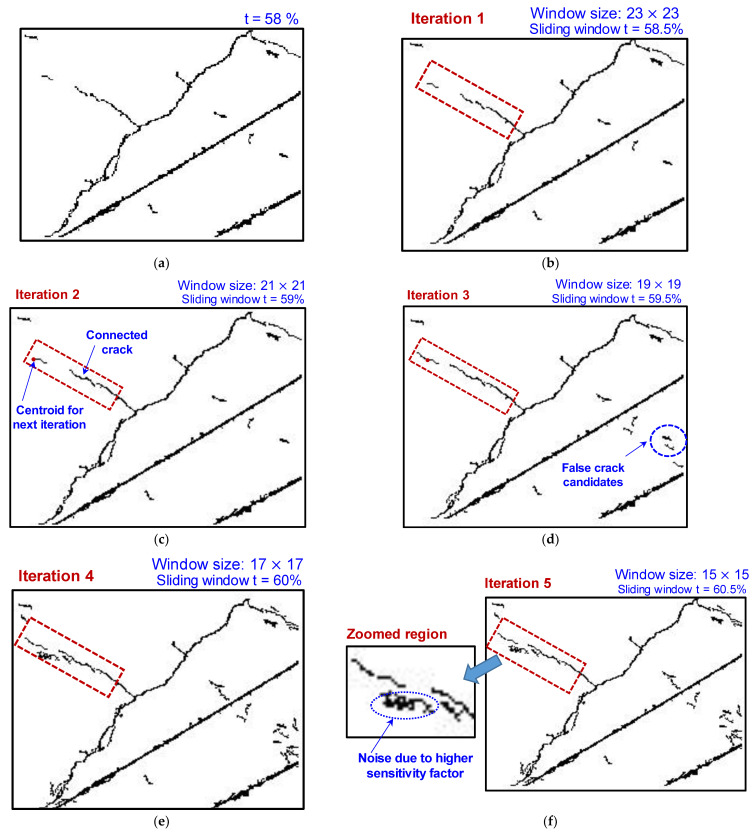
Results for identification of thin shallow cracks of single-pixel width. (**a**) Crack feature identification using 1D DL model. (**b**–**f**) Iteratively tracking missing segments of thin cracks (see (**b**)) post DL using a differential sliding window with increasing threshold.

With the proposed scheme, we tracked the missing thin shallow cracks on bridge piers from low-resolution UAV images, which are often undetected by DL models. Figure 6 displays a representative low-resolution image where the width of the crack is a single pixel. The iterative scheme shows promising results for tracking missing thin segments of cracks (Figure 7). This approach is superior to existing semantic segmentation for the following reasons: (a) although semantic segmentation predicts the location of a crack, the width of the crack region predicted by semantic segmentation is always greater than the crack width, and hence, the exact width of the crack in pixels cannot be quantified; (b) the proposed scheme does not require ground-truth labeling of thin cracks, whereas the semantic segmentation requires intensive labeling; (c) a deeper DL architectural layer is often required for metadata feature extraction when the crack signature is confined to a single pixel; however, such a scheme is unsuitable for real-time bridge inspections using UAVs with limited computational facilities.

**Figure 8:**
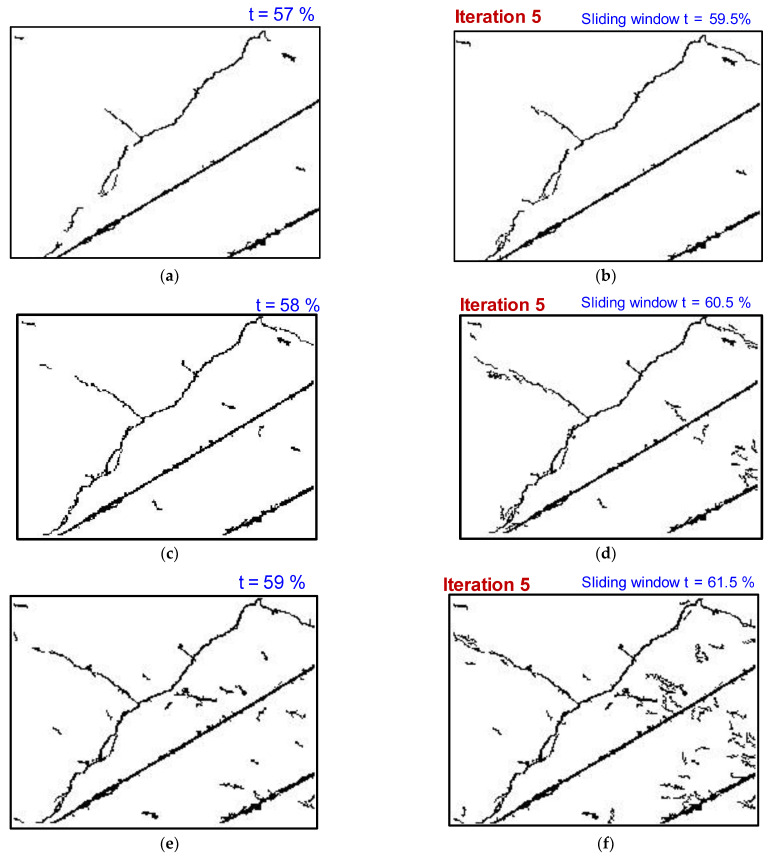
Effect of initial threshold percentage on tracking thin shallow cracks (**a**) t=57 %, (**c**) t=58 %, (**e**) t=59 %. (**b**,**d**,**f**) Tracking missing thin crack segments after 5^th^ iteration for corresponding pixelated residue with an initial threshold sensitivity parameters as shown in (**a**,**c**,**e**). Note: Δt for each iteration is 0.5 %.

**Figure 9:**
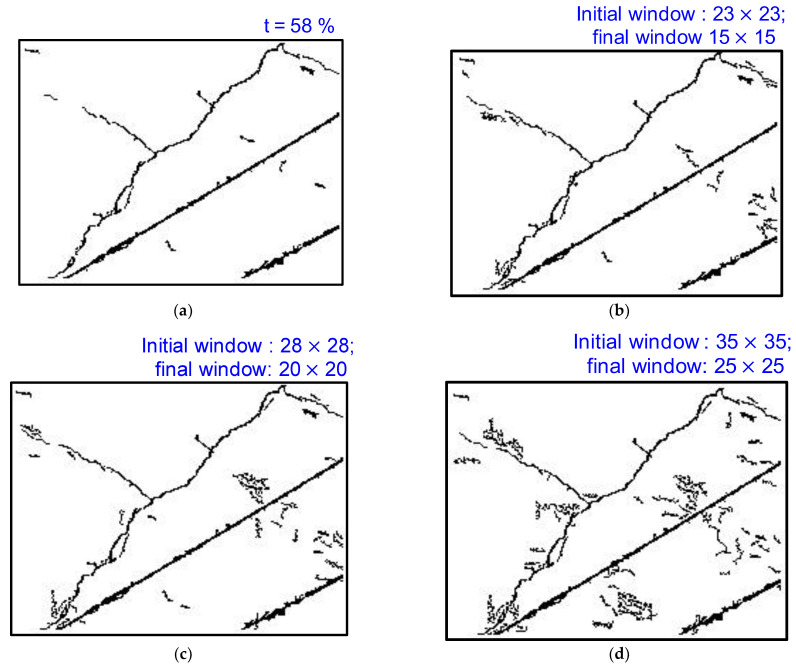
**(a)** Crack feature identification using 1D DL model with an initial sensitivity factor t=58 % during image preprocessing. Effect of initial window size (**b**) 23×23, (**c**) 28×28, and (**d**) 35×35 on tracking thin shallow cracks. The software and computational details of the desktop system employed for the crack detection scheme are as follows: Windows 10 operating system with Intel (R) Core (TM) i9-10900F CPU @2.80 GHz and 16 GB RAM memory. Various hidden processing steps to track missing shallow propagating thin crack segments in post-1D CNN-based DL are discussed in Section 3 (Figure 5). The computational time for the first iteration with the proposed algorithm (Figure 5) for an image as large as 3648 px × 5472 px is approximately 2–3 min. The computation time increases nonlinearly after each iteration. Wider segments of the crack are detected in the initial iterations, while thin shallow segments of the crack are detected in the subsequent iterations, which require a higher threshold that leads to additional features or disturbances that need to be filtered; hence, they are computationally expensive. A computational time comparison between the proposed framework and existing state-of-the-art DL models is presented in Table 1.

Despite the promising results, it is noteworthy to discuss the effects of various parameters on integral image binarization contributing to the final results, including (a) the effect of the initial threshold percentage (Figure 8) (b) the effect of the initial window size on tracking thin shallow cracks (Figure 9). Moreover, one limitation is that when the crack segment is close to the non-crack segment, the sliding window from the crack segment also searches for thin cracks or shallow features from the non-crack regime, which is undesired and requires additional filtering. This will be addressed in future studies. Another limitation of the proposed scheme is the misrecognition of disturbances, in addition to the detection of tiny cracks. Future work will focus on retaining the tiny cracks and filtering additional disturbances by combining the outputs of the DL model and the proposed research.

One of the limitations of image processing is the selection of predefined parameters. It is impossible to find a universal choice of parametric values that are versatile and suitable in all instances. Although the authors have used image processing with DL in our current work, we implemented the approach with a conservative choice of parameters, meaning that for image processing as a preprocessor, we chose a minimum threshold value that caters to a wide range of databases. We compensated for this minimal thresholding by incrementing the thresholding parameters in each iteration during image postprocessing on crack-classified segments from the DL.

**Figure 10:**
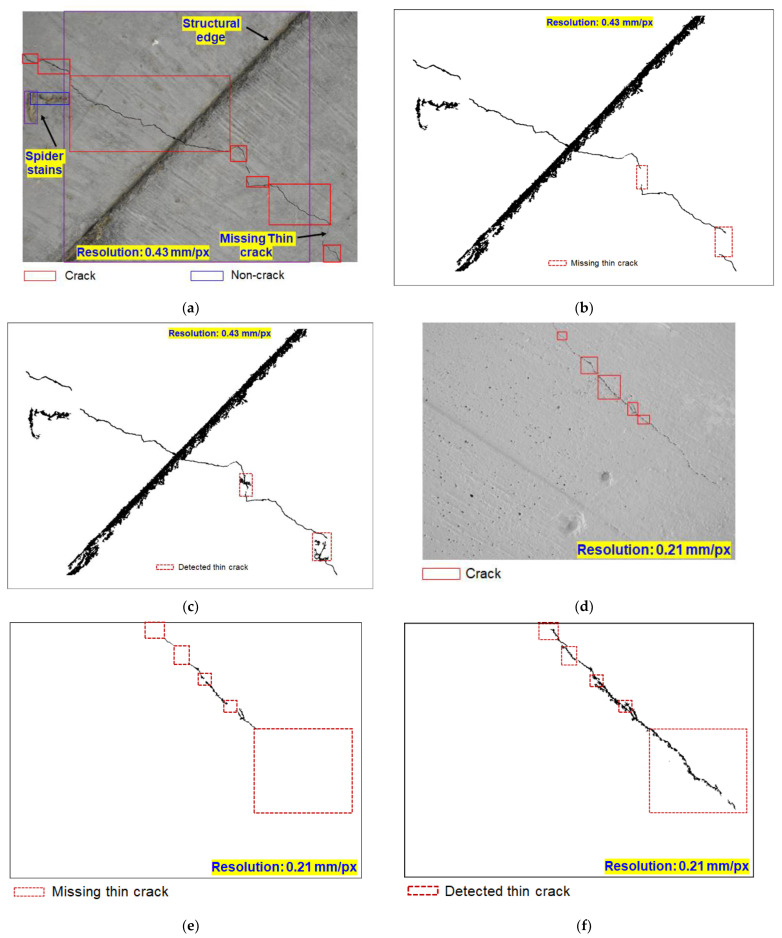
(**a**,**d**) Classification of crack in presence of non-crack features including structural edge, spider stains, peeling of concrete surface coating, stains and discoloration, and markings. (**b**,**e**) Representative pixelated results for crack and non-crack features shown in *(***a***,***d***)*, respectively. (**c**,**f**) Missing thin shallow cracks of single-pixel width (see *(***b***,***e**)) detected using DL-assisted image processing.

Figure 10 shows the results for crack and non-crack feature classification, and the subsequent detection of missing thin crack segments using the proposed scheme as discussed before. The proposed scheme of image-processing-assisted 1D-DFT-DL can detect cracks even when they are within the bounding box of non-crack features (Figure 10a,d). Pixelated results of crack and non-crack features are shown in Figure 10b,e. We further detected missing thin shallow propagating cracks of single-pixel width using 1D-DFT-DL-assisted image processing (Figure 10c,f)). The proposed approach could distinguish crack features from non-crack features including structural edges, spider stains, peeling of concrete surface coating, and structural markings. The pixel resolution of Figure 10a,b are 0.43 and 0.21 mm/px, respectively. We applied the proposed method on various pixel resolution images (mm/px), i.e., various widths of cracks of single-pixel size and the results are promising (Figure 10c,f). However, the proposed approach cannot distinguish high-stress-zone structural cracks from cracks appearing on the coating surface, which requires additional investigation. Few non-crack features whose major and minor axis are comparable are classified as background by the precursor image processing and are thus undetected during the proposed procedure.

**Figure 11:**
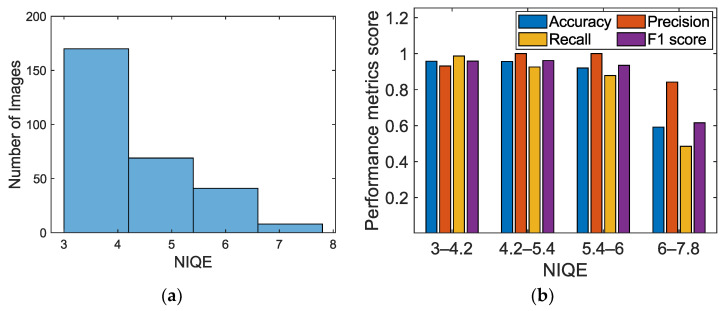
(**a**) Optical image quality that accounts for environmental uncertainty and optical variability of the database, quantified using natural image quality evaluation (NIQE) histogram. (**b**) Corresponding performance metrics scores for feature classification.

For the chosen database with a wide range of optical variabilities, environmental uncertainties, and surface texture variability, we quantified the quality of each image using a baseline-free indicator—natural image quality evaluation (NIQE) [69]. NIQE predicts deviations/artifacts in image statistics arising from optical and surface variability. Figure 11a presents the optical image quality quantified using the NIQE histogram. The quantitative range of the NIQE is 3–8; a low NIQE metric implies the best perceptual quality, with minimal undistortion. Ideally, we anticipate a reasonably high accuracy within the database uncertainty range or variability and a decrease for any unaccounted effects. However, the performance metrics deviate from the anticipated trend beyond the NIQE metric > 6 (Figure 11b) because of the skewed unbalanced dataset. The performance metrics of the proposed scheme are consistent for the NIQE range ≤ 6.

The proposed scheme is generic, can be adopted in a plug-and-play scheme, and can be employed regardless of the DL model used. A higher sensitivity/thresholding for precursor image processing increases the computational cost of DL. Higher sensitivity increases CCRs, which require additional detailed labeling prior to DL. Often, labeling with finer CCRs is misinterpreted and subjective to human interpretation. Moreover, for these types of problems, researchers have employed a deeper layer of architecture to extract hidden distinct features within a single-pixel width, thereby increasing the computational cost nonlinearly. However, the proposed scheme is computationally effective, wherein a cushion is provided to perform DL with a shallow architecture and extract missing information in an iterative manner post DL. Generally, the choice of the initial parameters in image processing determines the extent to which shallow cracks are determined. In addition, there can be a tradeoff between the choice of these parameters, which affects the computational cost and the sensitivity of crack detection using DL models; hence, the DL approach is sensitive to initial parameters such as the sensitivity factor in image processing. Accordingly, the proposed multi-threshold-based image processing, one as a precursor and the other as a postprocessor to the DL with a differential sliding window, is less sensitive to the initial handcrafted selections and predesigned parameters.

## 5. Summary

In the proposed approach, we first applied image binarization and DL-based crack identification. Subsequently, we detected thin propagating cracks that are often missed in DL-based classification by applying a differential sliding window of image processing to the end segments of cracks classified from the DL model. In general, a high initial threshold is used during image binarization as a precursor for identifying CCRs, resulting in an excessive amount of surface background and statistical features that are not associated with concrete surface features. The proposed approach provides a cushion or tradeoff for reducing the sensitivity parameters in the precursor, thereby reducing the potential CCRs and the associated computational costs during the training and testing of the DL model. The loss of sensitivity in the initial stage is compensated for during the image-binarization-based postprocessing. Hence, multi-threshold-based image processing as a precursor and postprocessor provides invariance in the framework to the choice of initial sensitivity parameters during image processing, hyperparameters, and the number of layers in the DL model. Meanwhile, semantic segmentation can result in a masked segmented region of a crack whose mask width is greater than that of a single-pixel propagating crack. The proposed approach enables image scalability with an efficient computational scheme while maintaining high accuracy in detecting thin shallow propagating crack segments.

The tracking of missing crack segments using differential sliding-window-based local image processing is equivalent to connecting crack segments based on the slope continuity information of the crack. However, in certain instances, the crack slope continuity fails when the direction of crack propagation is governed by the irregular orientation of the material microstructure and grains. Although the proposed scheme exhibited promising results, the overall approach is limited to the detection of thin crack segments or propagating cracks on the surface. Alternatively, physics-embedded-based NDI approaches combined with computer-vision-based sensing are required to detect the propagating cracks hidden within the structure. The proposed computer-vision- and DL-based approach inherently uses the geometrical information of cracks in concrete structures, wherein a sharp crack signature is followed by the tail of a thin shallow crack region or a propagating crack. This has potential applications in identifying real-time thin propagating cracks catering to a wide range of civil structures, wherein we employ UAVs or robotic setups with limited computational facilities to obtain low-resolution images owing to operating distance constraints.

## 6. Conclusions

The present study adopted multi-threshold image processing combined with a 1D frequency-domain DL model to classify cracks, including thin propagating shallow cracks with a single-pixel width. This approach effectively combines the advantages of (i) image binarization, (ii) computational effectiveness of a shallow 1D DL model, and (iii) inherent frequency-domain feature extraction characteristics of cracks in the Fourier-based DL model (1D-DFT-CNN). Specifically, the proposed scheme overcomes the limitations of (i) the fixed window size of sliding-window-based conventional CNNs and (ii) the time and effort required in pixelated labeling as employed in existing semantic segmentation. We demonstrated the proposed algorithm using low-resolution images obtained from different bridge piers using UAVs under different ambient lighting conditions and crack-like features.

## Figures and Tables

**Table 1:** Computational time comparison between the proposed framework and existing DL models.

Method	Training Time	Testing/Inference Time
Standard 2D-CNN (multi-pixel crack)	2 h 45 min 16 s	38–59 s/image [67]
Semantic segmentation	18 h	350 s/image [68]
Image processing, preprocessing, and post processing to 1D-DFT-CNN (proposed framework)	11 min 55 s (inclusive of preprocessing and DL)	Approx 0.02 s/image (60 images/s) (excluding image preprocessing and postprocessing)
		Approx 0.1–0.2 s/image (5–10 images/s) (including image preprocessing and DL testing)
		Approx 120–180 s/image (including image preprocessing, DL testing, and iterative image postprocessing)

## Data Availability

Data available on request due to restrictions from the funding agencies.
